# The Office Work and Stretch Training (OST) Study: An Individualized and Standardized Approach to Improve the Quality of Life in Office Workers

**DOI:** 10.3390/ijerph17124522

**Published:** 2020-06-23

**Authors:** Fabian Holzgreve, Laura Maltry, Jasmin Hänel, Helmut Schmidt, Andreas Bader, Markus Frei, Natalie Filmann, David Alexander Groneberg, Daniela Ohlendorf, Anke van Mark

**Affiliations:** 1 Institute for Occupational Medicine, Goethe-University Frankfurt, 60590 Frankfurt am Main, Germany; maltry@med.uni-frankfurt.de (L.M.); j.lampe@med.uni-frankfurt.de (J.H.); groneberg@med.uni-frankfurt.de (D.A.G.); ohlendorf@med.uni-frankfurt.de (D.O.); anke.van_mark@daimler.com (A.v.M.); 2 Daimler AG, 70171 Stuttgart, Germany; helmut.hs.schmidt@daimler.com (H.S.); andreas.b.bader@daimler.com (A.B.); 3 Mercedes-Benz AG, 76437 Rastatt, Germany; markus.frei@daimler.com; 4 Institute of Biostatistics and Mathematical Modeling, Goethe-University Frankfurt, 60590 Frankfurt am Main, Germany; filmann@med.uni-frankfurt.de

**Keywords:** occupational health, workplace health promotion, quality of life, stretching, musculoskeletal disorders, SF-36, Five-Konzept

## Abstract

In the context of workplace health promotion, physical activity programs have been shown to reduce musculoskeletal diseases and stress, and to improve the quality of life. The aim of this study was to examine the effects of using the “five-Business” stretch training device for office workers on their quality of life. A total of 313 office workers (173m/137f) participated voluntarily in this intervention–control study with an average age of 43.37 ± 11.24 (SD) years, 175.37 ± 9.35 cm in height and 75.76 ± 15.23 kg in weight, with an average BMI of 24.5 ± 3.81 kg/m^2^. The participants completed the stretch training twice a week for approximately 10 min for a duration of 12 weeks. The SF-36 questionnaire was used to evaluate the effectiveness of the intervention at baseline and after 12 weeks. Significantly improved outcomes in mental sum score (*p* = 0.008), physical functioning (*p* < 0.001), bodily pain (*p* = 0.01), vitality (*p* = 0.025), role limitations due to physical problems (*p* = 0.018) and mental health (*p* = 0.012) were shown after the stretching training. The results suggest that a 12-week stretching program for office desk workers is suitable to improve significantly their health-related quality of life.

## 1. Introduction

The progressive tertiarization of the economy sectors increases psychological demands and strains in the occupational setting [1]. In this context, increasing competition and rising productivity are leading to an increasing stress level, which in turn can have an impact on the health-related quality of life (QoL). The health-related QoL also correlates with sickness absenteeism and lower presenteeism [2,3]. Besides psychological components, physical complaints and diseases, such as musculoskeletal disorders (MSD), are also associated with health-related QoL [4,5]. For instance, chronic neck and shoulder pain lead to low work ability and poor quality of life [6]. MSD have great socioeconomic impact as they affect people’s wellbeing and welfare as well as reduce productivity [7]. Da Costa et al. [8], in a systematic review, identified that heavy physical work, smoking, high body mass index, high psychosocial work demands and the presence of comorbidity are risk factors with at least reasonable evidence for work-related MSD.

In Germany, MSDs are responsible for the 10 most important types of disease in disability days (20.9%), followed by sickness of the respiratory system (16.0%) and mental illness (15.2%) [9]. Since most diseases of the respiratory system are rather mild with an average case duration of 6.9 days, they are consequently rather short in comparison to MSD (19.7 days) and mental illness (37.0 days) [1]. In addition, the distribution of production downtime costs according to diagnosis groups was led by MSD (EUR 17.2 billion), followed by mental illness (EUR 12.2 billion) and respiratory system (EUR 10.6 billion) [10].

Employers seek to improve their employees’ health in order to reduce costs and increase productivity. One approach in occupational safety and health are workplace health promotion programs (WHPPs) [11]. Common approaches focus on nutrition [12], lifestyle [13], weight [14] or physical activity programs [15,16,17]. Predominantly, it has been the physical activity programs that have been shown to reduce MSD and stress and to improve the health-related QoL in both young and old workers [18,19,20,21]. In addition, there are investigations that physical activity can positively influence the state of mental illness [22,23]. Considering the effectiveness of reducing neck pain, strengthening exercises seem to be favored. For instance, in a systematic review performed by Sihawong et al. [24], strong evidence was found for the effectiveness of strengthening and endurance exercises in neck pain reduction. A recent meta-analysis of Louw et al. [25] revealed level II evidence for strengthening exercises in treating neck pain but not for improving QoL. Nevertheless, the authors concluded that the effect of endurance and stretching exercises needs to be explored further. In a recent review from Van Eerd et al. [26], moderate effects were found for stretching exercise programs and strong evidence for strengthening exercises in preventing upper extremity MSD.

However, to date, most studies have evaluated the effectiveness of stretching programs for MSD outcomes, only few have investigated the influence of such methods on the health-related QoL so far. Tunwattanapong et al. [15] conducted a regular stretching program, consisting of shoulder and neck stretching exercises, which were performed two times/day for five days/week over four weeks. They reported significant improvements in the pain visual analog scale, Northwick Park Neck Pain Questionnaire and Short-Form-36 questionnaire (SF-36). Stretching exercises lead to a reduced muscle stiffness of the musculotendinous unit and consequently, to an improved flexibility of both active and passive physiological structures [27]. This can reduce stress and/or pain [28,29]. Since the health-related QoL is associated with physical activity, MSD, stress and mental complaints, health-related QoL is a valid parameter with which to measure the effectiveness of WHPPs. In order to quantify the health-related QoL, the internationally widely used and standardized SF-36 questionnaire is appropriate, which has been applied to a number of different occupational groups [30,31] and inter alia to patients with low back pain [32] or psychiatric patients [33,34].

“Five-Konzept” is a new WHPP approach, which comprises a stretching health prevention program for office workers. Within 10 min, five specific trunk exercises were carried out on a special device (five-Business) at least two times a week. Further details can be found in Holzgreve et al. [35].

The aim of this study was to examine the effects of the “five-Business” stretch training for office workers on the health-related QoL. First, the baseline results of the SF-36 surveys of the intervention and control group were compared with the results of the German norm sample in order to obtain information on the general state of health of these office workers. Secondly, the effects of stretching training were examined in an intervention–control study using SF-36 outcomes.

## 2. Methods

### 2.1. Subjects

**Figure 1:**
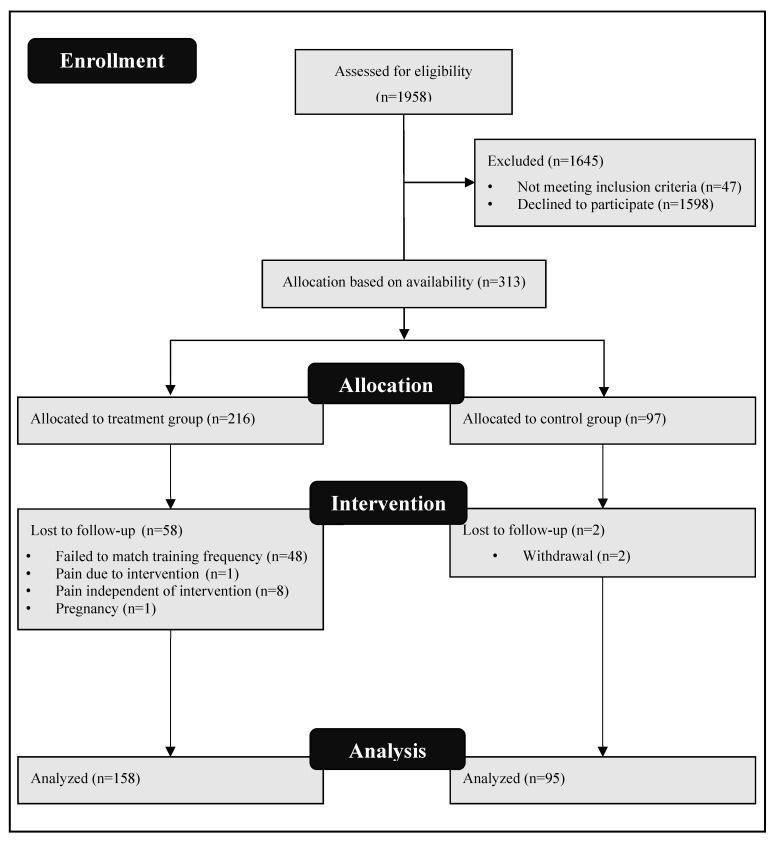
Disposition of study participants.

A total of 313 office workers (173m/137f) in one of several buildings of a large automotive company voluntarily participated in this intervention–control study (Figure 1). Moreover, 19.2% (35m/22f) of the participants of the intervention group had to terminate the study prematurely. Reasons for the dropout were most commonly because of the lack of regular participation in training due to business trips, the priority of work, part-time employment or private reasons (longer holidays, illness, pregnancy). Thus, 253 participants (138m/115f) successfully completed the study: 158 (102m/56f) in the intervention group (IG) and 95 (36m/59f) in the control group (CG) with 58 dropouts in IG and 2 dropouts in CG. Subjects were aged between 20 and 63 years (Figure 1; Table 1). Two months before the start of the study, 1958 office employees were contacted by email via the company’s internal health department. The aim was to reach healthy employees as well as employees with mild musculoskeletal complaints aged 18–65 years. All interested participants were contacted by telephone to clarify whether they met all the requirements and to arrange an appointment for the baseline testing. After baseline testing, the subjects were allocated to either the intervention or control group based on their availability. A total of 12.9% of the employees primarily contacted successfully completed the study (Figure 1).

Further socio-demographic characteristics can be taken from Table 1 on a gender-specific basis.

Exclusion criteria comprised relevant surgeries or surgical stiffening of the musculoskeletal system, relevant artificial joint replacement, severe diseases such as multiple sclerosis, myodystrophic or neurodegenerative diseases, congenital malpositions of the musculoskeletal system or an acute herniated disc. In addition, the intake of muscle relaxants or other drugs that influence the elasticity of the musculature, and pregnancy were considered as contra indicators. Participants met inclusion criteria when they were 18–65 years of age, worked in the office and were available during the offered training schedule. Further details can be found in the corresponding methodology article [35]. All participants signed written informed consent.

### 2.2. Intervention Program

**Figure 2:**
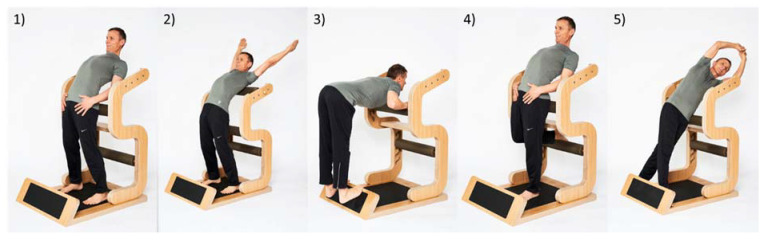
All five exercises of the “five-Business” workplace health promotion program. Exercises in the order of execution: (1) Stand, (2) Chest, (3) Ischio, (4) Hip and (5) Lateral.

The intervention program “five-Business” has been designed by the commercial provider Five-Konzept GmbH & Co. KG (Hüfingen, Germany) in cooperation with the Daimler health department for the implementation in company settings and for health promotion. The program comprises five stretch exercises of the trunk in two degrees of freedom on a specially developed device (Figure 2). All exercises can be performed on the device while standing and in business clothing. Height-adjustable cushions, which serve as abutments, allow the standardized program to be individually adapted. The participants complete the stretch training twice a week for approximately 10 min. Each exercise was held twice for 20 s. The intervention was scheduled for a duration of 12 weeks in which 22–24 training sessions were carried out. In order to meet the challenge of training on a regular basis, while still reflecting the operational reality of employees missing (e.g., due to vacation, business trips or sickness), the following guidelines were set: (1) the participants were allowed to be absent for a maximum of 2 weeks at a time; (2) after their absence, the participants were allowed to compensate for the missing training units with one additional unit per week; and (3), participants were allowed to miss a total of 2 out of the 24 training units.

In order to guarantee short walking distances to the training area, four devices in total were used: two on the first floor and two on the third floor of the four story building. The training area was screened off by partitions that were 1.60 m in height to ensure privacy. Both training areas were permanently supervised by an experienced trainer to guarantee the correct execution of the exercises. Missing participants were contacted via email to schedule a new appointment. The study was carried out between April and July 2018.

In terms of the training science, the “five-Business” program can be assigned to static stretching, since the musculature is statically stretched with continuous isometric contraction. The stretch exercises are whole body exercises, focusing on the trunk; they partly resemble yoga positions. The stretching was designed according to the course of the myofascial pathways, as stated by Myers [36] and include recommendations by McKenzie within the framework of the treatment concept in the trunk extension [37,38].

### 2.3. Short Form 36

The SF-36 version 1.3, developed by Ware and Sherbourne in 1992 in the United States [39], measures general health and health-related QoL taking into account physical, psychological and social factors. It is used to evaluate the individual patients health status, researching the cost-effectiveness of a treatment or for monitoring and comparing disease burden [40]. With 35 items, the SF-36 records eight dimensions of subjective health: *physical functioning* (PF), *role limitations due to physical problems* (RP), *bodily pain* (BP), *general health perceptions* (GH), *vitality* (VT), *social functioning* (SF), *role limitations due to emotional problems* (RE) and *mental health* (MH). The eight subscales can be assigned to two basic dimensions of subjective health: *physical* and *mental health* (PSC and MSC). The lower the score, the more disability [40] the subject possesses. The reliability of the German version of the SF-36 varies over the individual subscales between r = 0.67 and r = 0.85. In a study with back pain patients (n = 243), the internal consistencies for all subscales were determined (Cronbach’s α 0.60–0.93) [40]. In addition, 38 dichotomous questions on the health-related QoL were carried out as part of the survey.

### 2.4. Measurement Protocol

The general state of health using the health-related QoL survey SF-36 was evaluated before and after the intervention. The questionnaire was filled in on-site on a computer set up for this purpose. Subjects who could not be physically present were allowed to fill in the questionnaire online. The training was accompanied and controlled by trained trainer personnel throughout the intervention. The control group conducted the survey 12 weeks after baseline measurements analogous to the intervention group. The survey was used in April and July 2018.

### 2.5. Statistical Analysis

In order to compare baseline data, the results of the SF-36 subscales from the pre-test of the intervention and control group were compared with the results of the German norm sample from 1994 [40]. For this purpose, the standard data collection was filtered according to the following criteria: working full or part time (at least 15 h), employee with activity performed according to instructions, employee with independent performance in responsible position, employee with comprehensive management responsibilities, vocational training, polytechnic or university degree. The final norm sample consisted of 407 (244f/186m) subjects with a median age of 37 years.

The Kolmogoroff–Smirnoff–Lilliefors test was used to assess the normal distribution of the measured values. As almost all data were not normally distributed, nonparametric tests were used for all variables. In order to compare the baseline data of IG, CG and the German norm sample from 1994, the Kruskal–Wallis test was performed. Then, Conover–Iman comparisons with Bonferroni–Holm correction for multiple comparisons were performed. In addition, estimates of effects (eta^2^ = 0.01 small effect, 0.06 moderate effect, 0.14 strong effect) were calculated. For the statistical analysis within each group, the Wilcoxon matched pairs test for ordinal and the McNemar test for nominal data were performed. As a secondary analysis, the measured values between the intervention and control group by the Wilcoxon–Mann–Whitney U test were compared. The Fisher test was used to analyze nominally scaled values. Moreover, the respective effect sizes were calculated for each test. In order to test gender specific differences, the Wilcoxon–Mann–Whitney U test was used to identify significant gender-specific differences in IG. All tests were performed two sided, using a significance level of α = 5%. The statistics program “IBM SPSS Statistics 26” was used for the statistical evaluation.

### 2.6. Ethics Approval

All participants provided written informed consent to take part in the study in advance. This study was approved by the ethics research committee of the Medical Faculty of the Landesärztekammer Baden-Württemberg, Germany (F-2017-073).

## 3. Results

### 3.1. Comparison of SF-36 Baseline Data

**Figure 3:**
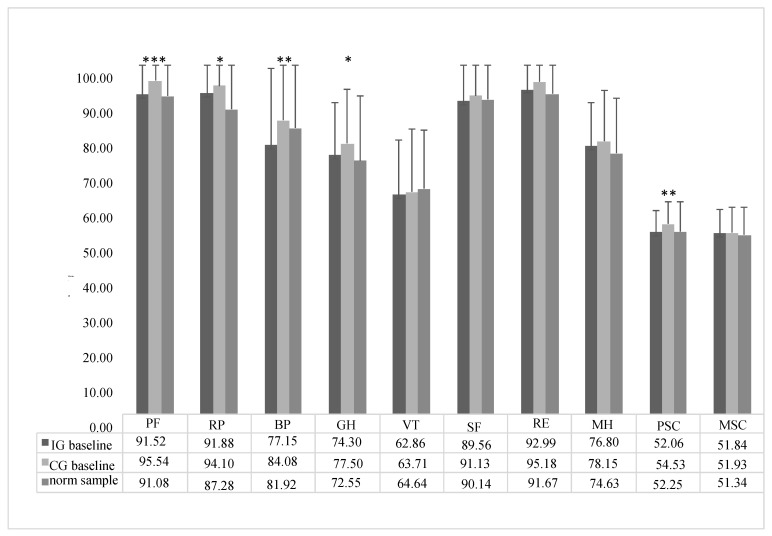
Baseline mean values of SF-36 outcomes for IG, CG and German norm data sample from 1994. Significant group differences are marked with asterisks. Subscales: physical functioning (PF), role limitations due to physical problems (RP), bodily pain (BP), general health perceptions (GH), vitality (VT), social functioning (SF), role limitations due to emotional problems (RE), mental health (MH) and physical and mental health sum scores (PSC and MSC, respectively). Significant differences are marked with asterisks (“*” = *p* < 0.05; “**” = *p* < 0.01; “***” = *p* < 0.001).

The comparison of the baseline SF-36 scores between IG, CG and the German norm sample from 1994 showed that CG had in each subscale a higher score than IG and, except for VT a higher score than the norm data sample (Figure 3). The Kruskal–Wallis test showed significant differences but, overall, small effect sizes between the baseline data in physical functioning (*p* < 0.001; eta^2^ = 0.037), role limitations due to physical problems (*p* = 0.047; eta^2^ = 0.01), bodily pain (*p* < 0.01; eta^2^ = 0.02), general health perceptions (*p* = 0.045; eta^2^ = 0.01) and physical health sum score (*p* < 0.001; eta^2^ = 0.014). All other comparisons were not significant. The *p*-values for direct comparisons of the respective significant subscales are shown in Table 2.

### 3.2. Examiation of the Effectiveness of the Intervention

**Figure 4:**
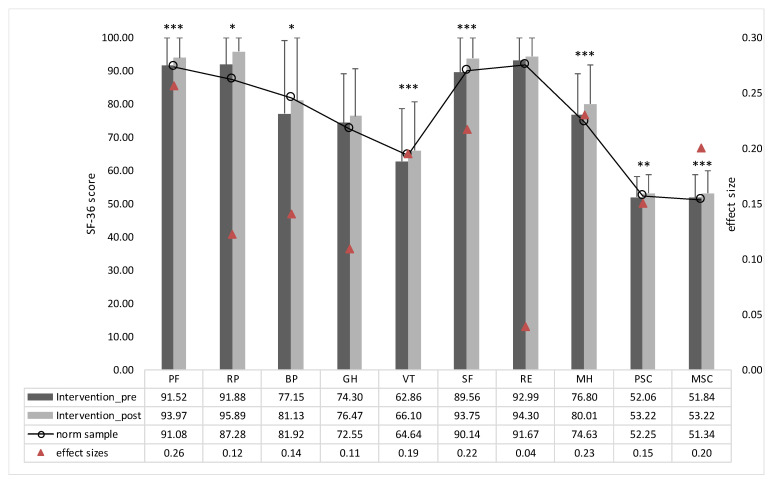
Mean values at baseline and after intervention for SF-36 outcomes of the intervention group. Mean values of the German norm sample of 1994 for SF-36 outcomes is also displayed. In addition, effect sizes are marked with red triangles. Significant pre–post differences are marked with asterisks. Subscales: physical functioning (PF), role limitations due to physical problems (RP), bodily pain (BP), general health perceptions (GH), vitality (VT), social functioning (SF), role limitations due to emotional problems (RE), mental health (MH) and physical and mental health sum scores (PSC and MSC, respectively). Significant differences are marked with asterisks (“*“ = *p* < 0.05;“**“ = *p* < 0.01;“ ***“ = *p* < 0.001).

The effectiveness of the intervention was examined by comparing the intervention group’s pre–post results; significant improvements were observed in physical functioning (*p* < 0.001), role limitations due to physical problems (*p* = 0.03), bodily pain (*p* = 0.013), vitality (*p* < 0.001), social functioning (*p* < 0.001) and mental health (*p* < 0.001) (Figure 4). All subscales show improvements in the score compared to the baseline, although the overall effect sizes were rather small (0.04–0.26). While the baseline scores of the intervention group in the subscales bodily pain, vitality and social functioning were lower than those of the German norm sample, the intervention led to overall significant improvements in vitality and social functioning (Figure 4). Due to the significant increase in bodily pain, the IG is post-interventional within the range of the norm. Both the physical sum score and mental sum score of IG showed a significant improvement (PSC: *p* = 0.009; MSC: *p* < 0.001) compared to the baseline values (Figure 4). The outcomes of the subscales and sum scores of the baseline comparison within IG are summarized in Table 3.

The control group showed no significant improvements compared to the baseline values. No directional change was descriptively discernible (Table 3).

**Figure 5:**
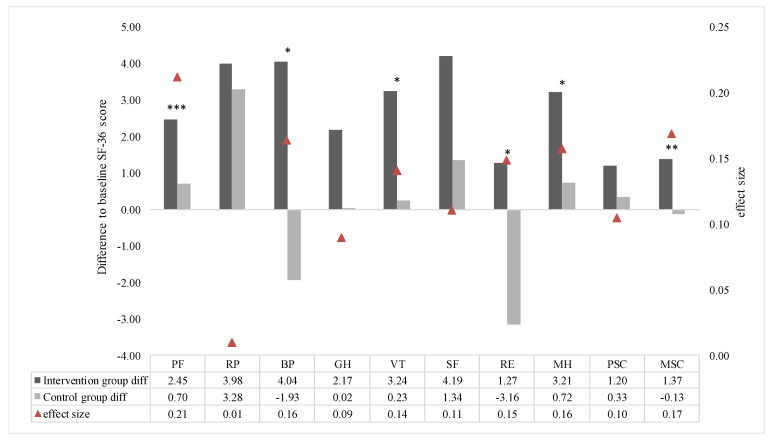
Mean pre–post differences for IG and CG in SF-36 outcomes. Effect sizes are marked with red triangles. Significant pre–post differences are marked with asterisks. Subscales: physical functioning (PF), role limitations due to physical problems (RP), bodily pain (BP), general health perceptions (GH), vitality (VT), social functioning (SF), role limitations due to emotional problems (RE), mental health (MH) and physical and mental health sum scores (PSC and MSC, respectively). Significant differences are marked with asterisks (“*” = *p* < 0.05; “**” = *p* < 0.01; “***” = *p* < 0.001).

The effects of the intervention control study are shown in Figure 5. Pre–post differences of IG and CG were compared. A significant difference can be observed for the subscales physical functioning (*p* < 0.001), bodily pain (*p* = 0.010), vitality (*p* = 0.025), role limitations due to emotional problems (*p* = 0.018) and mental health (*p* = 0.012). For the subscales role limitations due to physical problems, general health perceptions and social functioning no significant differences could be determined (Figure 5). In each SF-36 outcome, a greater improvement was observed in IG. The overall effect sizes are, analogous to baseline comparisons of IG, small. The comparison of the physical and the mental sum score show significant differences for MSC (PSC: *p* = 0.103; MSC: *p* = 0.008) in the control group (Figure 5). The effect sizes had rather small outcomes in all scales, in the range of 0.01 and 0.21 (Figure 5). Overall, outcomes of the subscales and sumscores between IG and CG are summarized in Table 3.

The participants of the intervention group felt significantly less irritable (*p* = 0.031), their energy faded significantly less fast (*p* = 0.041) and worries kept them up significantly less at night (*p* = 0.039).

### 3.3. Analysis for Gender Effects

The analysis for gender dependent differences showed no significant effects in any subscale of the SF-36 within IG. 

## 4. Discussion

The main aim of this study was to measure the effectiveness of physical stretch training by means of a questionnaire on the health-related quality of life (QoL). The results of the intervention–control comparison relieved that significant improvements occurred in both areas due to the training. In three out of four psychological scores (*mental health* (*p* = 0.012), *role limitations due to emotional problems* (*p* = 0.018) and *vitality* (*p* = 0.025)) and two out of four physical scores (*physical functioning* (*p* < 0.001) and *bodily pain* (*p* = 0.01)) significant improvements were determined, thus indicating that stretch training may not only affect physical health parameters, but also mental health. These results confirm the initial hypothesis and are in line with studies reporting associations between physical activity and mental health and health-related QoL, respectively [18,19,20,21].

Looking at the cumulative scores, it is shown that only the *mental sum score* (*p* = 0.008) was significant improved while the *physical sum score* of SF-36 (*p* = 0.103) was not.

As well-known [41], the psychological well-being could be positively influenced, e.g., by less pain (*p* = 0.009) and a resulting improvement in social participation.

In addition, the intervention seems to encourage the subjects to influence their own well-being and to let physical complaints be controlled (RE (*p* = 0.018)). Furthermore, the reasons for the increased mental well-being may lie in an increased range of motion and thus reduced pain. This hypothesis can be supported by significant improvements in *bodily pain* and *physical functioning,* which can be confirmed by recent studies [42,43,44]. Goncalves and colleagues [43] found an inverse association between the frequency and severity of neck pain and the global health-related QoL. Other studies have shown that back pain and, in particular, chronic low back pain are associated with higher levels of subclinical anxiety and depression [42,44]. Stretching is one of the many methods that are recommended when suffering MSD, especially back pain [26]. In most cases, pain associated with MSD causes some degree of disability due to the limitation of range of motion [45,46]. Therefore, stretching may improve MSD-related pain by increasing the range of motion, improving blood circulation within the affected musculature, or by improving the nutrition of the intervertebral discs. Lawand et al. [47] reported of significant findings in SF-36 characteristics bodily pain, role limitations due to emotional problems, physical functioning, vitality, mental health, using the global postural reeducation stretching method in patients with chronic low back pain. In this study, 61 patients with chronic low back pain underwent a weekly 60-min stretching session for 12 months. These results confirm the results of the current study as exactly the same SF-36 characteristics showed significant improvements after a stretching period of 12 weeks, indicating that stretching training also leads to QoL benefits in office workers.

The study of Tunwattanapong et al. [15] is the only one, so far, that has evaluated health-related QoL outcomes in a stretching WHPP. They reported improvements in the health-related QoL (physical sum score—*p* < 0.001; mental sum score—*p* = 0.084). In contrast to the results of Tunwattanapong et al. [15], the present study showed significant improvements in the *mental sum score*, but not in the *physical sum score*, however, in two of the four measured dimensions. This could be explained by differences in the study protocol concerning exercise choice, intervention duration or sessions per week; the choice of exercises is distinctly different. While Tunwattanapong et al. [15] focused on exercises for the neck and shoulders, the present study mainly selected exercises for whole muscle chains; these are similar to yoga exercises. Hartfiel et al. [48] demonstrated that workplace-specific yoga training can contribute to the reduction of back pain and stress, as well as to the improvement of well-being. In the study of Tunwattanapong et al. [15], mainly mobilization exercises were used for the neck area, whereas in this study whole-body stretching exercises were used. However, both studies have shown a clear improvement in their respective scores, even if they were not significant, underlining the importance of stretching exercises on the health-related QoL. Furthermore, Tunwattanapong’s study and the current study have shown that stretching in the operational setting appears to influence the health-related QoL.

A descriptive comparison of the absolute values between the two groups shows that, despite weaker values before the start of the study, the scores in all mental characteristics are after the intervention higher in the intervention group. This fact applies to all mental and not to any physical characteristics (Table 3). The heterogeneous composition of both groups provides one explanation. The intervention group is significantly older than the control group (*p* < 0.010), due to the fact that it was a waiting control group. This circumstance could explain the lower level of physical characteristics (Table 1). Despite the lower level of physical characteristics, the subjects of the intervention group achieved significant improvements in three out of four physical characteristics.

The analysis for gender specific differences suggests that gender does not appear to have any influence on the expression of SF-36 characteristics. According to this, women and men react similarly to stretching on both a physical and psychological level [49].

However, when interpreting the results, in view of the overall low effect sizes, it must be taken into account that the effects of stretching on characteristics of the health-related quality of life are supposedly on the threshold of clinical relevance despite significant differences. On the other hand, the effect size as a measure of changes in psychology is controversial and in a test with such good power as the SF-36 we could speak of moderate effects as early as 0.1 to 0.2. In particular, when the small group sizes are taken into account, the significance appears to be a good measure for the inclusion of the success of the intervention.

A random allocation would have led to a significant reduction in the number of participants and would have threatened the feasibility of the study, which is why a quasi-control group was chosen. Nevertheless, the lack of randomization must be taken into account when interpreting the results. For example, factors such as long holidays or absence, high workload or illness, which favor allocation to the control group, might influence the quality of life. Further possible uncontrolled sources that influence mental stabilization could be the individual attention of the trainers in the accompanied training units. Not least the mere possibility of participating in such a complex measure, in the sense of appreciation and experiencing a positive corporate culture, could also have had a positive effect on the result. Several studies have shown that especially active over passive pauses have positive effects on shoulder/neck pain [50,51,52]. It is noticeable that the reported frequency of breaks is much higher than in this study. It cannot be excluded that two breaks of 10 min per week will have an effect, but it is not to be expected.

Minor amendments had to be carried out on the method (Holzgreve et al. [35]): Two training sessions per week could not be adhered to due to holidays, meetings and illness, so that the study protocol had to be adapted (see Section 2.2). Further limitations can arise in the practical application if the training is not carried out with this high personnel expenditure, or that the extreme attention by sports scientists/physiotherapists during the training could also have contributed considerably to the effect. Since a successful implementation of a WHPP depends on the willingness of employees to participate, future studies should evaluate whether stretch training programs without such a high personnel expenditure achieve comparable results. Furthermore, future studies should observe whether a random assignment and equal pauses in the control group lead to similar results.

## 5. Conclusions

These results suggest that a stretching program performed for three months can improve the health-related QoL for office desk workers. The greatest changes were observed at the level of mental health. These findings indicate that a physical intervention program not only affects physical health parameters, but also mental health. The effects of such a WHPP go beyond the musculoskeletal system, indicating a promising measure to cover the current challenges of increasing competition and productivity demands in the workplace.

## Figures and Tables

**Table 1:** Baseline demographic characteristics of participants in the intervention and the control groups. Significant differences in the baseline between IG and CG are marked with asterisks.

	Entire Sample [Mean (SD) or %]			Intervention Group [Mean (SD) or %]			Control Group [Mean (SD) or %]		
		Male	Female		Male	Female		Male	Female
**Initial participants**	n = 313	n = 172	n = 137	n = 216	n = 135	n = 78	n = 97	n = 38	n = 59
**Dropouts**	n = 60	n = 35	n = 22	n = 58	n = 33	n = 22	n = 2	n = 2	n = 0
**Final participants**	n = 253	n = 138	n = 115	n = 158	n = 102	n = 56	n = 95	n = 36	n = 59
Age (years) **	43.37 (11.24)	46.74 (10.21)	39.32 (11.11)	44.94 (10.56)	47.42 (9.52)	40.33 (10.84)	40.83 (11.92)	44.86 (11.86)	38.37 (11.37)
**Height (cm)**	175.37 (9.35)	180.34 (7.18)	169.41 (8.10)	176.21 (8.76)	180.78 (6.24)	167.89 (6.21)	173.98 (10.15)	179.11 (9.36)	170.85 (9.38)
Weight (kg) ***	75.76 (15.23)	82.90 (13.51)	67.12 (12.52)	78.15 (15.02)	83.83 (13.19)	67.43 (12.49)	71.97 (14.74)	80.25 (14.23)	66.83 (12.64)
BMI (kg/m^2^) **	24.50 (3.81)	25.45 (3.64)	23.35 (3.71)	25.03 (3.97)	25.64 (3.74)	23.90 (4.18)	23.61 (3.35)	24.89 (3.34)	22.81 (3.13)
**Handedness (% right)**	94.90	94.20	95.70	93.70	94.10	92.90	96.80	94.20	98.30
**Doing sports (% yes)**	72.70	72.50	73.00	70.90	70.60	71.40	75.80	77.80	74.60
**Smoking (% non-smoker)**	88.90	90.60	87.00	89.90	92.20	85.70	96.80	86.10	88.10
h/sports/week ***	3.13 (3.01)	3.2 (3.17)	3.04 (2.80)	2.77 (3.17)	2.97 (3.29)	2.41 (2.92)	3.96 (2.45)	4.04 (2.56)	3.90 (2.40)

Cross-gender test for baseline differences between IG and CG. *p* < 0.01 = **; *p* < 0.001 = ***.

**Table 2:** *p*-Values for baseline data differences of IG, CG and the German norm data sample from 1994 for significant SF-36 outcomes PF, RP, BP, MH and PSC. Bonferroni–Holm correction for multiple comparisons has been applied.

	IG-CG	IG-Norm Sample	CG-Norm Sample
PF	< 0.001	0.005	0.001
RP	> 0.05	> 0.05	0.023
BP	0.049	< 0.000	> 0.05
GH	> 0.05	> 0.05	0.039
PSC	0.021	0.021	> 0.05

**Table 3:** SF-36 outcomes (mean, SD, *p*-value and effect size) are shown for IG (pre–post), CG (pre–post) and intervention control study (pre–post differences). Diff. means difference between post and baseline. Subscales: physical functioning (PF), role limitations due to physical problems (RP), bodily pain (BP), general health perceptions (GH), vitality (VT), social functioning (SF), role limitations due to emotional problems (RE), mental health (MH) and physical and mental health sum scores (PSC and MSC, respectively).

	Intervention Group				Control Group				Intervention–Control Study			
	Mean (SD)				Mean (SD)				Mean (SD)			
	Pre	Post	*p* Value ^a^	Effect Size r ^b^	Pre	Post	*p* Value ^a^	Effect Size r ^b^	diff. IG	diff. CG	*p* Value ^c^	Effect Size r ^b^
**PF**	91.52 (10.38)	93.97 (10.17)	<0.001	0.26	96.51 (6.92)	97.22 (6.79)	0.056	0.19	2.45 (8.09)	0.70 (5.18)	<0.001	0.21
**RP**	91.88 (21.97)	95.89 (14.07)	0.030	0.12	94.15 (18.85)	97.19 (11.51)	0.053	0.01	3.98 (23.09)	3.28 (17.29)	>0.2	0.01
**BP**	77.15 (21.97)	81.13 (19.62)	0.013	0.14	83.68 (20.61)	81.76 (17.98)	>0.2	0.15	4.04 (18.69)	−1.93 (18.55)	0.01	0.16
**GH**	74.30 (14.88)	76.47 (14.04)	0.053	0.11	77.61 (15.35)	77.67 (13.41)	>0.2	0.08	2.17 (12.08)	0.02 (12.24)	0.154	0.09
**VT**	62.86 (15.77)	66.10 (14.67)	<0.001	0.19	63.74 (17.91)	63.96 (16.45)	>0.2	0.13	3.24 (11.88)	0.23 (13.94)	0.025	0.14
**SF**	89.56 (15.32)	93.75 (12.03)	<0.001	0.22	91.13 (14.70)	92.63 (13.08)	>0.2	0.10	4.19 (13.21)	1.34 (14.57)	0.080	0.11
**RE**	92.99 (19.26)	94.30 (19.25)	>0.2	0.04	95.44 (16.57)	92.28 (21.44)	0.090	0.13	1.27 (22.61)	−3.16 (19.49)	0.018	0.15
**MH**	76.80 (12.39)	80.01 (11.85)	<0.001	0.23	78.02 (14.34)	78.75 (13.27)	>0.2	0.14	3.21 (10.27)	0.72 (10.70)	0.012	0.16
**PSC**	52.06 (6.19)	53.22 (5.46)	0.009	0.15	54.11 (5.24)	54.42 (4.18)	>0.2	0.09	1.20 (5.24)	0.33 (4.64)	0.103	0.10
**MSC**	51.84 (6.84)	53.22 (6.62)	<0.001	0.20	52.00 (7.37)	51.75 (7.32)	>0.2	0.15	1.37 (5.86)	−0.13 (5.59)	0.008	0.17

^a^ Wilcoxon test. ^b^ Effect size r after Rosenthal: 0.1 “small effect”, 0.3 “moderate effect”, 0.5 “strong effect”. ^c^ Wilcoxon–Mann–Whitney U test.

## Abbreviations

SD	standard deviation
QoL	quality of life
MSD	musculoskeletal disorders
WHPPs	workplace health promotion programs
IG	intervention group
CG	control group
PF	physical functioning
RP	role limitations due to physical problems
BP	bodily pain
GH	general health perceptions
VT	vitality
SF	social functioning
RE	role limitations due to emotional problems
MH	mental health
PSC	physical sum score
MSC	mental sum score
